# Psychological assessment in 355 Chinese college students with androgenetic alopecia

**DOI:** 10.1097/MD.0000000000011315

**Published:** 2018-08-03

**Authors:** Xia Wang, Chunping Xiong, Li Zhang, Bin Yang, Rongfang Wei, Liqian Cui, Xiangbin Xing

**Affiliations:** a Department of Dermatology, the First Affiliated Hospital of Jinan University; b Department of Dermatology, the First Affiliated Hospital of Guangzhou Medical University; c Department of Dermatology, the First People's Hospital of Lanzhou City, Lanzhou; d Dermatology Hospital of Southern Medical University, Guangzhou, P.R. China; e Department of Gastroenterology, the First Affiliated Hospital of Sun Yat-sen University.

**Keywords:** androgenetic alopecia, checklist-90-R, college students, psychological assessment

## Abstract

Although the association of the psychological problems and androgenetic alopecia (AGA) gained the increasing attention, the psychosocial state in college students with AGA remains unknown. We recruited a total number of 355 college students with AGA from 18 universities in Southern China for interview. The Symptom Checklist-90-R (SCL-90-R) survey was used to assess the psychological state of these students. There were significant differences in somatization, obsessive–compulsive, interpersonal sensitivity, depression, phobic anxiety, psychoticism, and global severity index (GSI) between college students with AGA and the controls. Moreover, regarding the impact of specialty, scores for the interpersonal sensitivity, depression, and phobic anxiety in medical students and art students with AGA were significantly higher than other professions. In addition, obsessive–compulsive and GSI in art students with AGA were significantly higher compared with other professions. These findings suggested that the therapeutic approach for the psychological problems should be considered in the tailored treatment for AGA in the college students.

## 1 Introduction

Androgenic alopecia (AGA), or man pattern hair loss, is the most common form of alopecia in both men and women.^[1]^ AGA is a slowly progressive form of alopecia which begins after the initiation of puberty.^[2]^ Male pattern baldness is a physiologic process that was significantly associated with androgens and occurred in genetically predisposed hair follicles.^[3]^ Nyholt et al^[4]^ put forward that heredity is the major contributing factor to AGA, and makes up 80% of the variance. AGA can occur in men even with the normal levels of androgen, and has the clinical manifestation of hair loss genetically related to the individual.^[3]^

Hair plays an important role in human social and sexual communication. Individuals suffering from AGA often have the feeling that alopecia is a serious condition probably causing unfavorable consequences in their life. Adolescents and young adults including college students are at the important stage of the psychological development and their psychological situation is unstable.^[5]^ It is possible that college students with AGA are confronted with the challenge of additional stress of hair loss and this could be a heavy burden on their coping abilities. However, the psychosocial state in college students with AGA remains largely unknown. Therefore, we conducted this study to assess the mental health of AGA college students in Southern China using the SCL-90-R scales.

## 2 Materials and methods

### 2.1 Study design

This study was designed as a cross-sectional study. The study protocol was reviewed and approved by the institutional ethical committee of Guangzhou Medical University. Written informed consent form was obtained from all participants. The study was carried out in collaboration with Department of Psychiatry in Guangzhou Medical University.

Three hundred and fifty-five college students with AGA and 406 healthy controls were enrolled. After signing an informed consent form, the participants were assigned to a face-to-face interview in a quiet and comfortable room. All participants completed Italian version of the Symptom Check List-90-Revised (SCL-90-R).

### 2.2 Selection of participants and healthy control subjects

The participants with AGA were collected from 18 universities in Guangdong Province located in Southern China. All participants were willing to attend a psychosomatic dermatology consultation were considered for enrollment in the study. The inclusion criteria are as follows: the age between 17 and 40 years; the diagnosis of AGA was based on the following considerations: progression of hair loss, family history of thinning and visual confirmation of a receding frontal hairline, and the presence of thin and short hairs in the frontal area and vertex; the use of a magnifying glass or dermoscopy may assist diagnosis. Neurotic alopecia, scalp disease, and certain chemicals and radiation induced alopecia were not present.

For the clinical classification of AGA, the Norwood classification system,^[6]^ a standard classification scheme which has been shown to have good test–retest reliability, was used. Men with “female pattern hair loss” (not involving the frontal hairline) and women were assessed separately using the Ludwig classification system.^[7]^ We defined AGA according to 3 degrees of the severity, normal to mild (Norwood types I and II, Ludwig types I), moderate (Norwood types III, IV, and V, Ludwig types II), and severe (Norwood types VI and VII, Ludwig types III). Data from 355 patients with AGA were analyzed according to this classification system.

### 2.3 Psychological questionnaires

The symptom Checklist-90-Revised (SCL-90-R; Derogatis, 1994),^[8,9]^ which was used to assess psychological distress, is a 90-item self-report symptom with 83 covering the following dimensions: somatization, obsessive–compulsive, interpersonal sensitivity, depression, anxiety, hostility, phobic anxiety, paranoid ideation, and psychoticism. It is rated on a 5-point Likert scale of distress, ranging from “not at all = 0” to “extremely = 4.” The 9 symptom dimensions were divided into 3 global indexes, namely, the global severity index (GSI), positive symptom total (PST), and positive symptom distress index (PSDI). The GSI, the mean score of all items, is considered to be the best representation of an overall psychological distress dimension. To ensure the obtainment of the complete data, 2 Graduate Research Assistants checked all data prior to each participant's departure. Over all, there were no data missed in this study.

### 2.4 Statistical analysis

We used *t* test, one-way analysis of variance (ANOVA) and chi-square test for the statistical analysis using SPSS 16.0 (IBM Corp, Armonk). The Tukey test was also used to assess the difference between any 2 groups posterior to one-way ANOVA analysis. After the significance of the chi-square test, the adjusted residuals test and the Gamma test were carried out to locate the associations. Statistical significance was set at *P* < .05.

## 3 Results

Three hundred and fifty-five college students with AGA (340 men, 15 women, age range 17 and 40 years) were enrolled in this study. The sample included 130 undergraduate students, 92 graduate students, 76 Ph.D. candidates, and 57 post doctorates from 18 universities in Guangdong Province located in Southern China. Four hundred and six healthy subjects who were comparable to the study group in terms of age, sex, education, and geographic data were included in the control group. Demographical data including age, sex, and education status of all participants were recorded.

Comparison of psychological measurements between AGA patients and control subjects are shown in Table 1. There were significant differences in terms of somatization (*P* < .001), obsessive–compulsive (*P* < .001), interpersonal sensitivity (*P* < .001), depression (*P* < .001), phobic anxiety (*P* < .001), psychoticism (*P* < .001), and global severity index (GSI) (*P* < .001) between college students with AGA (n = 355) and controls (n = 406). The differences in other subscales between the 2 groups were not statistically significant. Equally interesting, regarding the association of specialty of the college students and AGA, scores for the interpersonal sensitivity (*P* < .001), depression (*P* < .001), and phobic anxiety (*P* < .001) in medical and art students with AGA were significantly higher than other professions. Furthermore, obsessive–compulsive and GSI in art students with AGA were significantly higher than other professions. In addition, scores for somatization, anxiety, and psychoticism in literature students were significantly higher than other professions. The detailed results are shown in Table 2. Moreover, Table 3 indicated that somatization, interpersonal sensitivity, depression, anxiety, phobic anxiety, psychoticism, and GSI were significantly associated with the educational history in college students with AGA. In addition, after the significance of the chi-square test, we performed the Gamma test to analyze the association between education level and AGA types (contingency coefficient = 0.457, *P* < .001).

**Table 1:**
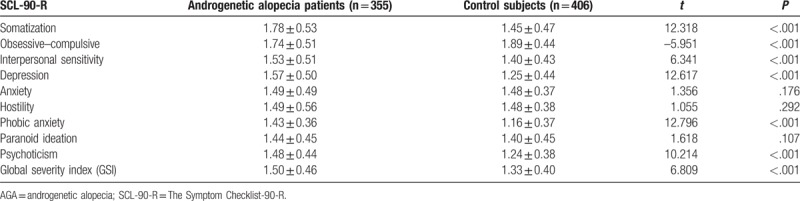
Comparison of psychological status between college students with AGA and healthy controls.

**Table 2:**
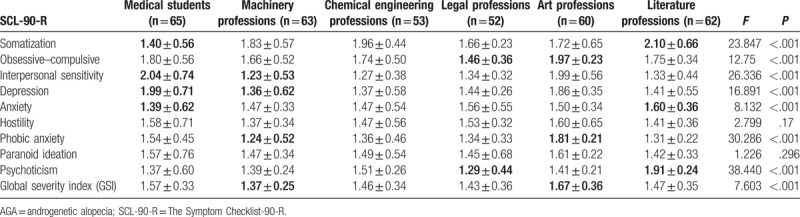
Impact of specialty on psychological status among college students with AGA.

**Table 3:**
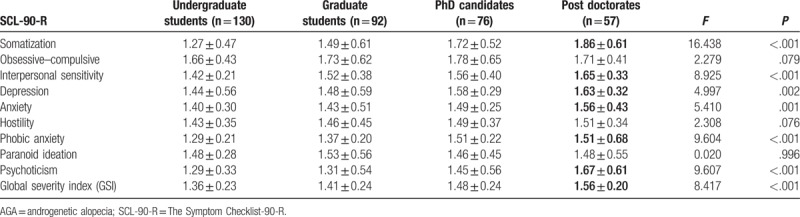
Impact of educational history on psychological status among college students with AGA.

## 4 Discussion

In this study, we assessed for the first time the psychological state in Chinese college students with AGA. The college students with AGA exhibited the increased levels of somatization, obsessive–compulsive, interpersonal sensitivity, depression, phobic anxiety, psychoticism, and GSI as compared with the control subjects. It's known that hair is an important facet of human appearance that is commonly used for recognition and is one determinant of physical attractiveness.^[10]^ Depression present in these students may be related to the feelings of low self-confidence caused by the alteration of their appearance. Somatization in the college students with AGA may be associated with their depression and phobic anxiety. These results suggested that students feel hard to deal with the presence of hair loss which has a significant impact on the psychological status, quality of life, and social interaction.

By comparing the impact of specialty on psychological status of students with AGA using SCL-90-R, we found that medical students and art students with AGA have psychological situation significantly worse than other professions in terms of interpersonal sensitivity, depression, hostility, phobic anxiety, and paranoid ideation. Doctors are more prone to anxiety, depression, drug and alcohol problems, and even suicide due to the factor of specialty.^[2,10,11]^ Medical students are under pressure from sources associated with medical training, leading to mental health problems to a certain degree.^[12]^ Bramness et al^[13]^ reported that the medical students exhibited a lower level of self-esteem as compared with the general population, while the difference between the mental health of the students and the control group did not reach the statistical significance. Similarly, our study indicated that the students in health profession with normal presence of hair have a little higher score in interpersonal sensitivity, depression in comparison with other professions using SCL-90-R, while the difference was not statistically significant. Therefore, medical students might be prone to mental health problems when a heavier burden and stress caused by the presence of AGA. Moreover, art students are more frequently exposed to the public, they would be more concerned about their outlook. Hair loss might thus put higher pressure on these art professional students in daily life, and lead to a higher score in obsessive–compulsive, hostility, phobic anxiety, paranoid ideation, and GSI in these students.

Recent studies show that AGA is multi-factor related diseases. Age, occupation, mental status, genetic, endocrine, and other factors were related to AGA onset.^[14–17]^ The majority of AGAs occur at the age of <40 years and many patients exhibited this disease at the age of <30 years. Giltay et al^[18]^ found that a greater degree of unfavorable psychological impact of hair loss in younger men and those who have the earlier onset of hair loss. Physical appearance is extremely important to most young men, and early onset of hair loss can have a negative effect on self-image and self-esteem.^[19]^ The impairment of self-confidence makes it difficult to find appropriate life partners and employment. The findings in this study may provide new perspectives for clinicians in the treatment of college students with AGA. The therapy of college students with AGA should include the approaches for their psychological problems caused by AGA, in addition to those approaches for the hair loss.

## Author contributions

**Conceptualization:** Bin Yang.

**Data curation:** Li Zhang.

**Formal analysis:** Rongfang Wei.

**Methodology:** Liqian Cui.

**Resources:** Chunping Xiong.

**Software:** Rongfang Wei, Liqian Cui.

**Writing – original draft:** Xia Wang.

**Writing – review and editing:** Xia Wang, XiangBin Xing.
